# Pharmacologic depletion of border-associated macrophages worsens disease in a mouse model of meningitis

**DOI:** 10.1186/s40478-025-02126-5

**Published:** 2025-09-23

**Authors:** Susanne Dyckhoff-Shen, Ilias Masouris, Hans-Walter Pfister, Stefanie Völk, Sven Hammerschmidt, Matthias Klein, Uwe Koedel

**Affiliations:** 1 Department of Neurology, LMU University Hospital, LMU Munich, Munich, Germany; 2 Department of Molecular Genetics and Infection Biology, Interfaculty Institute for Genetics and Functional Genomics, University of Greifswald, Greifswald, Germany; 3 Department of Neurology, Klinikum Grosshadern of the University of Munich, Marchioninistr. 15, D-81377 Munich, Germany

**Keywords:** Streptococcus pneumoniae, Mouse model of pneumococcal meningitis, Macrophages, Clodronate liposomes, Pneumolysin, Caspase-1

## Abstract

Pneumococcal infection of the leptomeninges triggers a strong inflammatory response, contributing to tissue damage and adverse outcome in meningitis. While border-associated macrophages (BAM) are thought to initiate immune responses against pathogens, their exact role in pneumococcal meningitis (PM) - especially at later stages - remains unclear. This study examined the impact of BAM depletion on disease progression. Mice received intracisternal injections of clodronate liposomes (CL) to deplete BAM, followed three days later by intracisternal infection with *Streptococcus pneumoniae*. At 18 h post-infection, CL-treated mice exhibited clinical signs similar to controls treated with phosphate-buffered saline liposomes (PBSL). However, CL-treated mice had lower cerebrospinal fluid leukocyte counts, increased expression of brain immune mediators, and elevated plasma levels of neuronal damage (NEFL) and astrocyte activation (S100B) markers. Over a 42-h observation period - during which ceftriaxone therapy was started 18 h post-infection - CL-treated mice showed significantly worse outcomes: 9 of 12 reached termination criteria versus 1 of 9 PBSL-treated mice. This correlated with more severe neuropathology, higher bacterial loads, and persistent inflammation. Notably, infection with a pneumolysin-deficient mutant conferred strong protection against disease aggravation caused by macrophage depletion, whereas caspase-1 inhibition - despite its known immunosuppressive effects in experimental PM - did not. These findings underscore a critical immunoregulatory role for BAM in PM, particularly in resolving rather than initiating inflammation. Their absence exacerbates disease severity, mainly due to increased bacterial proliferation and elevated levels of bacterial toxins.

**Supplementary Information:**

The online version contains supplementary material available at 10.1186/s40478-025-02126-5.

## Introduction

Bacterial meningitis remains a major global health challenge despite the availability of effective antibiotic therapy. It ranks among the ten leading causes of death from infectious diseases and is the fourth most common cause of disability-adjusted life years (DALYs) - a composite measure of years lost due to ill health, disability, or premature death - attributable to neurological disorders worldwide [1, 2]. In Europe, *Streptococcus pneumoniae* (*S. pneumoniae*, the pneumococcus) is the predominant etiological agent, accounting for approximately two-thirds of all cases [3–5]. In industrialized countries, pneumococcal meningitis (PM) continues to be associated with high morbidity and mortality. The case fatality rate remains around 10%, and up to 50% of survivors suffer long-term neurological sequelae [3–7].

The pathogenesis of PM involves a profound inflammatory response within the leptomeningeal compartment, which substantially contributes to neuronal injury and poor clinical outcomes [3, 4, 8]. This inflammatory cascade is initiated by the recognition of pneumococcal pathogen-associated molecular patterns by pattern recognition receptors, particularly Toll-like receptors [9], expressed on resident central nervous system (CNS) cells. Among these, resident macrophages are currently considered the principal immune sentinels in the leptomeninges and key producers of pro-inflammatory cytokines following pneumococcal invasion [10, 11]. However, experimental evidence supporting this concept is limited and, in some cases, inconsistent.

To date, three studies have examined the consequences of CL-induced depletion of BAM in experimental PM models, all focusing on the acute phase (within 24 h post-infection) [12–14]. Collectively, these studies demonstrated that macrophage depletion increases bacterial burden in the brain, but they reported inconsistent effects on the associated inflammatory response. Pinho-Ribeiro et al. [12] described reduced meningeal leukocyte counts after CL treatment in mice, although closer inspection of the data suggested only limited infection-driven leukocyte infiltration, with reductions largely reflecting decreases relative to CL-treated uninfected controls. Polfliet et al. [13] observed diminished cerebrospinal fluid (CSF) pleocytosis in rats but paradoxically elevated cytokine and chemokine levels (notably interleukin [IL]-6 and CXCL2), whereas Trostdorf et al. [14] reported no major changes in CSF leukocytes or cytokines in rabbits. Together, these discrepant findings highlight important advances in linking resident macrophages to early infection control, but they also underscore critical uncertainties: how macrophage depletion shapes the local inflammatory response, leukocyte dynamics, and neuropathological changes such as vasogenic edema, as well as how it influences neuroinflammation and brain pathology at later stages of disease.

The present study addresses these gaps by investigating the role of resident macrophages in the progression of brain inflammation and pathology during both early stages and - importantly, for the first time - advanced stages of experimental PM.

## Materials and methods

### Mouse model of PM

We used a well-characterized mouse model of PM as described previously [9, 15, 16]. Briefly, mice were weighed, body temperature was measured using a rectal probe, and motor activity was assessed using the open field test (OFT). A physical examination, including clinical scoring, was then performed. The clinical scoring comprised a beam walk test, a postural reflex test, and assessments of piloerection, vigilance, and/or presence of seizures. The maximum clinical score was 13, indicating moribund mice that required euthanasia for ethical reasons; a score of 0 indicated uninfected, healthy mice. Following the clinical assessment, mice received subcutaneous buprenorphine (WDT, #02540, Germany) for analgesia, which was reapplied every 8 h to maintain its effect. One hour later, bacterial meningitis was induced via transcutaneous puncture of the cisterna magna, followed by injection of 15 µl of *S. pneumoniae* type 2 (D39 strain; 10⁷ colony-forming units [CFU]/ml in PBS) using an Omnican^®^ 100 insulin syringe under short-term isoflurane anesthesia. After awakening, the mice were returned to their cages and clinically reevaluated. Clinical scores were assessed at 18 h (time of ceftriaxone administration), 24 h, and 42 h post-infection. At the end of the experiment, mice were anesthetized with ketamine/xylazine. After local anesthesia with lidocaine, a skin incision was made to expose the skull. A catheter was then inserted into the cisterna magna through a hole drilled at the caudal end of the occipital bone to collect CSF for white blood cell (WBC) counts. Blood samples were collected via cardiac puncture to determine bacterial titers and to obtain plasma for the analysis of selected cytokines and biomarkers of CNS cell activation or injury. Subsequently, animals were transcardially perfused with ice-cold heparinized PBS. The brain was removed, and the cerebellum was dissected and homogenized in 1 ml sterile PBS for bacterial quantification. The remaining brain tissue was immediately frozen in tissue freezing medium (Leica Biosystems) for cryosectioning and subsequent histopathological and molecular analyses.

### Experimental groups in the mouse model

A total of 78 male C57BL/6n mice (12–20 weeks old, weighing 24–30 g, with no mean weight differences between groups) were used in these experiments. We performed the study exclusively in male C57BL/6 mice due to the higher reproducibility of the PM model in males in our hands, and in line with the principle of animal reduction as mandated by the European Directive 2010/63/EU.

To deplete BAM [17], mice received trancutaneous intracisternal (i.c.) injections of 15 µl CL on two consecutive days. Control animals received phosphate-buffered saline-filled liposomes (PBSL; both Liposoma Technology). This regimen was chosen based on pilot experiments comparing the efficiency of single versus double CL administration (see last paragraph of this section), which revealed slightly higher depletion rates with the double-dosing protocol. Three days after the second liposome administration, mice were infected i.c. with *S. pneumoniae* D39.

To assess the role of BAM in the early phase of PM, CL- and PBSL-treated mice were evaluated 18 h post-infection (*n* = 6 per group). Mice that received i.c. injections of PBS instead of *S. pneumoniae* and were evaluated 18 h post-injection served as healthy controls (*n* = 3), in order to illustrate meningitis-associated inflammatory and pathological alterations.

To study macrophage function during disease progression, the observation period was extended to 42 h post-infection, requiring antimicrobial treatment to reduce mortality from overwhelming infection. Ceftriaxone (100 mg/kg, intraperitoneally [i.p.]) was administered at 18 and 24 h post-infection. Groups for this extended observation included PBSL-treated infected mice (*n* = 9) and CL-treated infected mice (*n* = 12). PBS-injected mice evaluated at 42 h post-injection served as healthy controls (*n* = 3).

To further investigate the mechanisms underlying adverse outcomes after macrophage depletion, additional CL-treated infected mice were either administered VX-765, a selective caspase-1 inhibitor (50 mg/kg i.p.) immediately before infection and again at 18 h post-infection, or infected with an isogenic pneumolysin (PLY)-deficient *S. pneumoniae* D39 mutant (*n* = 8 per group, D39Δply). Macrophage-depleted mice infected with wild-type D39 served as positive controls for these interventions (*n* = 10), and also as an internal validation cohort for the initial 42-hour observation study.

In pilot experiments to confirm effective macrophage depletion, mice received either single or double i.c. injections of CL or PBSL (*n* = 2 and 3 per group, respectively). CD206-positive cells, representing meningeal and perivascular macrophages, were quantified in four brain sections containing lateral ventricles and hippocampal tissue 72 h after the final injection. Following double administration, CL reduced meningeal and perivascular macrophage numbers by 82% and 80%, respectively (see **Supplemental Figure**), whereas a single i.c. injection achieved lower depletion rates of 74% and 70%. We also compared the phenotype of CL-treated mice that received an i.c. injection of PBS with PBS-injected controls after a 42-hour observation period. No differences were observed in clinical status (e.g., score: 0 ± 0 in both CL-treated healthy controls and untreated healthy controls), CSF leukocyte counts (417 ± 104 vs. 367 ± 58 cells/µl, respectively), or brain pathology (no bleedings detectable in either group; brain albumin concentration: 45.3 ± 7.8 vs. 50.3 ± 12.4 ng/mg brain protein, respectively). Therefore, experiments with an 18-hour observation period were omitted in accordance with the principle of reducing animal numbers as mandated by the European Directive 2010/63/EU.

### Determination of bacterial titers in blood and brain

Blood samples and cerebellar homogenates were serially diluted in sterile saline, plated on blood agar plates, incubated for 24 h at 37 °C with 5% CO₂, and colonies were counted.

### Analysis of cerebral bleeding

Frozen brains were sectioned coronally at 10 μm thickness using a cryostat. Starting from the anterior region of the lateral ventricles, eight serial sections were collected at 0.3 mm intervals throughout the ventricular system and photographed using a digital camera. Hemorrhagic spots were counted, and the total bleeding area per slice was quantified using ImageJ (NIH).

### Immunohistochemical analysis of murine brains

Coronal brain sections (10 μm thick) containing the lateral ventricles and hippocampal tissue were stained with one or two of the following primary antibodies: a polyclonal goat anti-mouse CD206/MMR antibody (AF2535, R&D Systems), a monoclonal rat anti-mouse CD31/PECAM1 antibody (553379, BD Pharmingen™), a polyclonal rabbit anti-pneumococcal antibody (generated by the Hammerschmidt lab), or a polyclonal rabbit anti-mouse albumin antibody (600-401-254, Rockland). To block non-specific binding, sections were incubated with 0.5% bovine serum albumin (BSA) prior to antibody application. Sections were then incubated with the respective primary antibodies for 2 h at room temperature (RT) at the following dilutions: 1:80 for CD206, and 1:100 for CD31, anti-pneumococcal, and anti-albumin antibodies. Fluorescent labeling was performed using secondary antibodies conjugated to Alexa Fluor 488 or Alexa Fluor 594, specific to goat, rat, or rabbit IgG, applied at a 1:200 dilution for 1 h at RT. Nuclei were counterstained with 4′,6-diamidino-2-phenylindole dihydrochloride (DAPI) at a 1:10,000 dilution. After mounting with Vectashield H-1000 medium, images were acquired using an Olympus BX-51 fluorescence microscope equipped with an Olympus DP28-CU color camera. Image analysis was conducted using Olympus cellSens Standard software by two independent, blinded observers, and results were averaged.

### Measurement of IL-1β, IL-6, albumin, NEFL, S100B and TREM2 concentrations in brain homogenates and/or blood plasma samples

Concentrations of selected inflammatory mediators and biomarkers of cell activation or injury in brain and/or plasma samples were determined by ELISA (DY401 and DY406 combined with DY008B from R&D Systems; ab207620 from Abcam; RDR-NEFL-Mu and RDR-S100B-Mu from Reddot; MBS4503107 from MyBiosource) according to the manufacturers’ instructions.

### mRNA expression analyses of murine brains

Total RNA from murine brain sections was extracted using Aurum Total RNA Mini Kit (Bio-Rad, 7326820) according to manufacturer’s instructions. Reverse transcription into cDNA was achieved using the iScript cDNA Synthesis Kit (Bio-Rad, 1708890) according to manufacturer’s instructions. Quantitative PCR (qPCR) was performed on a qTower^3^ Real-Time PCR Detection System (Analytikjena) using the SsoAdvanced Universal Probes Supermix (Bio-Rad, 1725280) and PrimePCR™ SYBR^®^ Green assays (UniqueAssayIDs: CCL2: qMmuCED0003785, CXCL2: qMmuCED0050757, ICAM1: qMmuCED0048485, GFAP: qMmuCID0020163, GAPDH: MmuCID0018612) and a custom-made PrimePCR™ SYBR^®^ Green array containing 30 inflammatory cytokines and receptors, and 2 different housekeeping genes detailed on **Supplemental Table**. GAPDH was used as reference gene. The custom array was performed in duplicate (for groups with *n* ≥ 6) using pooled cDNA from three randomly selected mice per group.

### Statistical analysis

Prior to project implementation, sample size planning was performed under the supervision of a statistician, and statistical analysis methods were predefined. Planning was based on CSF WBC count, a validated parameter in PM that serves as a diagnostic marker, reflects the immune response, and is suitable for detecting pharmacological or genetic interventions. Sample size calculation indicated six animals per group. Between 18 and 42 h post-infection, 4/6 CL-treated animals reached termination criteria, compared with 0/6 in the PBSL group. Following the up-and-down procedure and the principles of animal reduction mandated by European Directive 2010/63/EU, group sizes were increased by *n* = 6 (CL) and *n* = 3 (PBSL). Statistical analysis confirmed a significant difference in the predefined primary endpoint. Due to the substantial disease burden, no further experiments were conducted. Six animals per group were used to investigate mechanisms underlying adverse outcomes after macrophage depletion, with group sizes subsequently increased by 2 and 4 animals, respectively, following the up-and-down procedure in accordance with the principles of animal reduction. Non-infected controls (*n* = 3, intrathecal PBS instead of *S. pneumoniae*) served as technical controls for the injection procedure and had been validated in previous studies. Results for key parameters such as CSF WBC count were consistent with earlier findings; therefore, no additional controls were included. Since the comparison between healthy and meningitis animals served only to demonstrate disease-typical alterations, no significant differences to healthy controls are shown in the figures. Statistical analyses and graph generation were performed using GraphPad Prism software. The principal statistical test was an ANOVA with Newman Keuls multiple comparison test or a log-rank test (Mantel) for survival. Differences were considered significant at *p* values < 0.05. Data are given as mean ± standard deviation (SD).

## Results

### Pharmacological macrophage depletion only mildly affects the development of PM

**Fig. 1:**
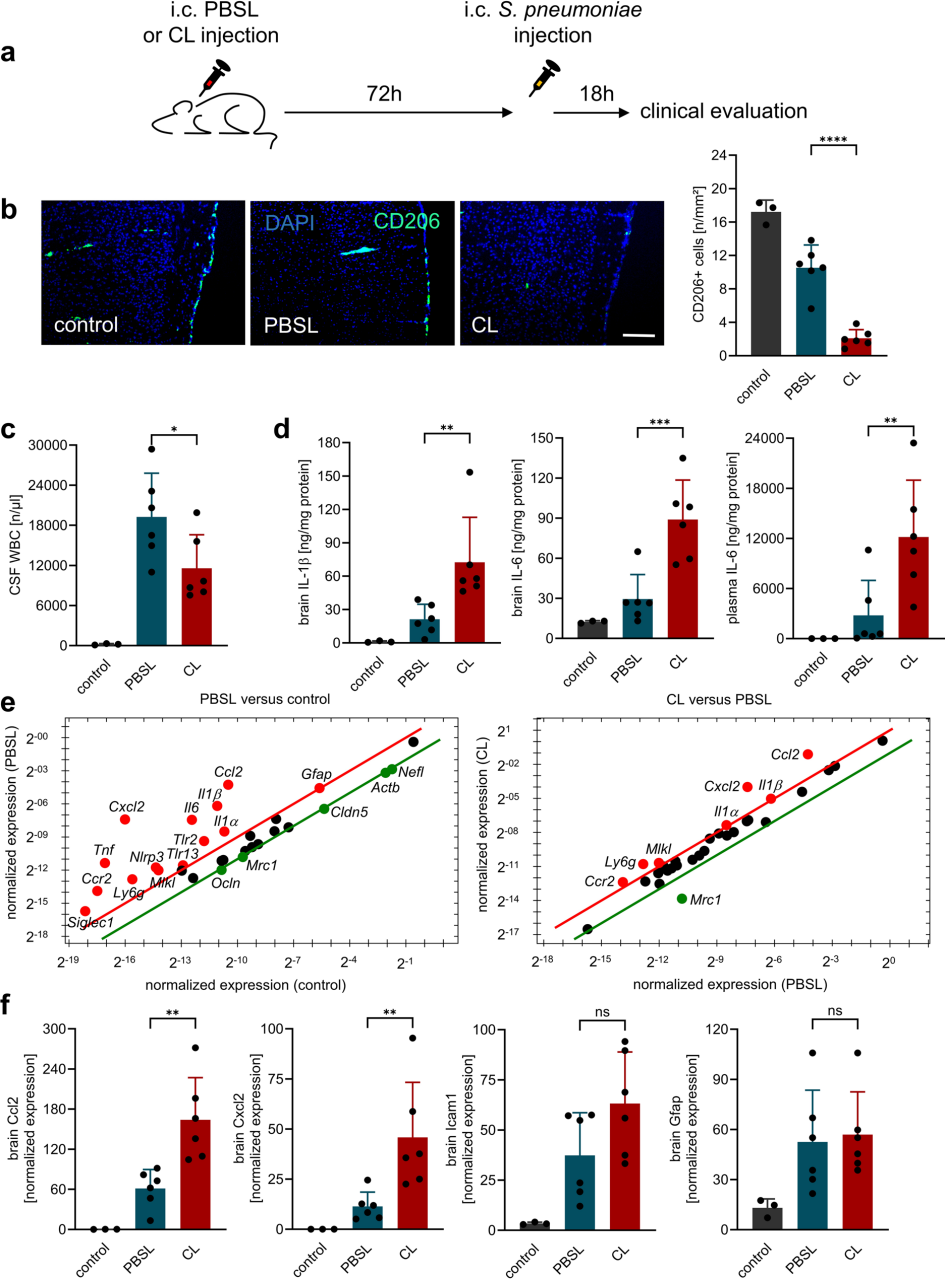
BAM depletion alters early inflammatory responses in PM. (**a**) Experimental design: Intracisternal (i.c.) administration of clodronate liposomes (CL) or PBSL liposomes was performed on two consecutive days. Seventy-two h later, mice were infected by i.c. inoculation with live *S. pneumoniae* and clinically assessed 18 h post-infection (*n* = 6 per group). Control animals only received an intracisternal injection of PBS (*n* = 3). (**b**) Quantification of CD206⁺ meningeal macrophages by immunohistochemistry (scale bar = 100 μm) showed an ~ 90% reduction in CL-treated animals compared to healthy controls, whereas PM alone resulted in an ~ 40% reduction in macrophage numbers in PBSL-treated mice. (**c**) CSF pleocytosis was significantly reduced in CL-treated mice (~ 40% decrease), indicating impaired leukocyte recruitment. (**d**) Protein levels of IL-1β and IL-6 in brain homogenates (and plasma) were significantly elevated in CL-treated infected mice compared to PBSL-treated infected mice. (**e**) PrimePCR™ array analysis revealed upregulation of proinflammatory mediators (e.g., IL-1β, CCL2, CXCL2) and downregulation of MRC1 (CD206) in CL-treated mice. (**f**) RT-PCR confirmed higher expression levels of CCL2 and CXCL2 in CL-treated mice; ICAM-1 and GFAP expression remains unchanged. Data represent mean ± SD; * *p* < 0.05, ** *p* < 0.01, *** *p* < 0.001, **** *p* < 0.0001 (ANOVA and Newman Keuls multiple comparison test). Since the comparison between healthy and meningitis animals served only to demonstrate disease-typical alterations, no significant differences to healthy controls are shown in the figures

BAM were traditionally thought to initiate immune responses against pathogens in the leptomeningeal space [10]. However, experimental evidence supporting this concept is limited and not convincing. Therefore, we first checked the impact of macrophage depletion on the development of meningitis that means the first 18 h post-infection. Similar to earlier studies [12–14], i.c. administration of CL proved to be an effective method for reducing CD206-positive BAMs, namely meningeal and perivascular macrophages. In our model, their numbers decreased by approximately 90% and 88%, respectively, compared with healthy controls (Fig. 1). Notably, mice with PM alone already showed an approximately 40% reduction in these cell populations compared with healthy controls.

Macrophage depletion led to a significant reduction in CSF pleocytosis (by 40%). Interestingly, this was associated with increased expression of cytokines and chemokines in the CNS: We observed significantly elevated protein levels of IL-1β and IL-6 in the brains of CL-treated infected mice compared to PBSL-treated infected controls. Accordingly, significantly higher levels of IL-1β and IL-6 were detected in the CSF of CL-treated than PBSL-treated infected mice (IL-1β: 3,701 ± 1,999 vs. 957 ± 693 pg/ml; IL-6: 44,923 ± 16,156 vs. 11,041 ± 2,805 pg/ml; *p* < 0.01 and *p* < 0.001, respectively). Both cytokines were absent in control CSF. In our PrimePCR™ assay investigations, we detected an increased expression of seven out of 30 analyzed factors, including IL-1β, CCL2, and CXCL2, in CL-treated mice compared to PBSL-treated mice, while Mrc1 (CD206) was, as expected and consistent with the depletion results, significantly downregulated. Subsequent RT-PCR analyses confirmed our assay results: CL-treated meningitis animals showed higher expression levels of CCL2 and CXCL2 (but not GFAP) compared to PBSL-treated mice.

**Fig. 2:**
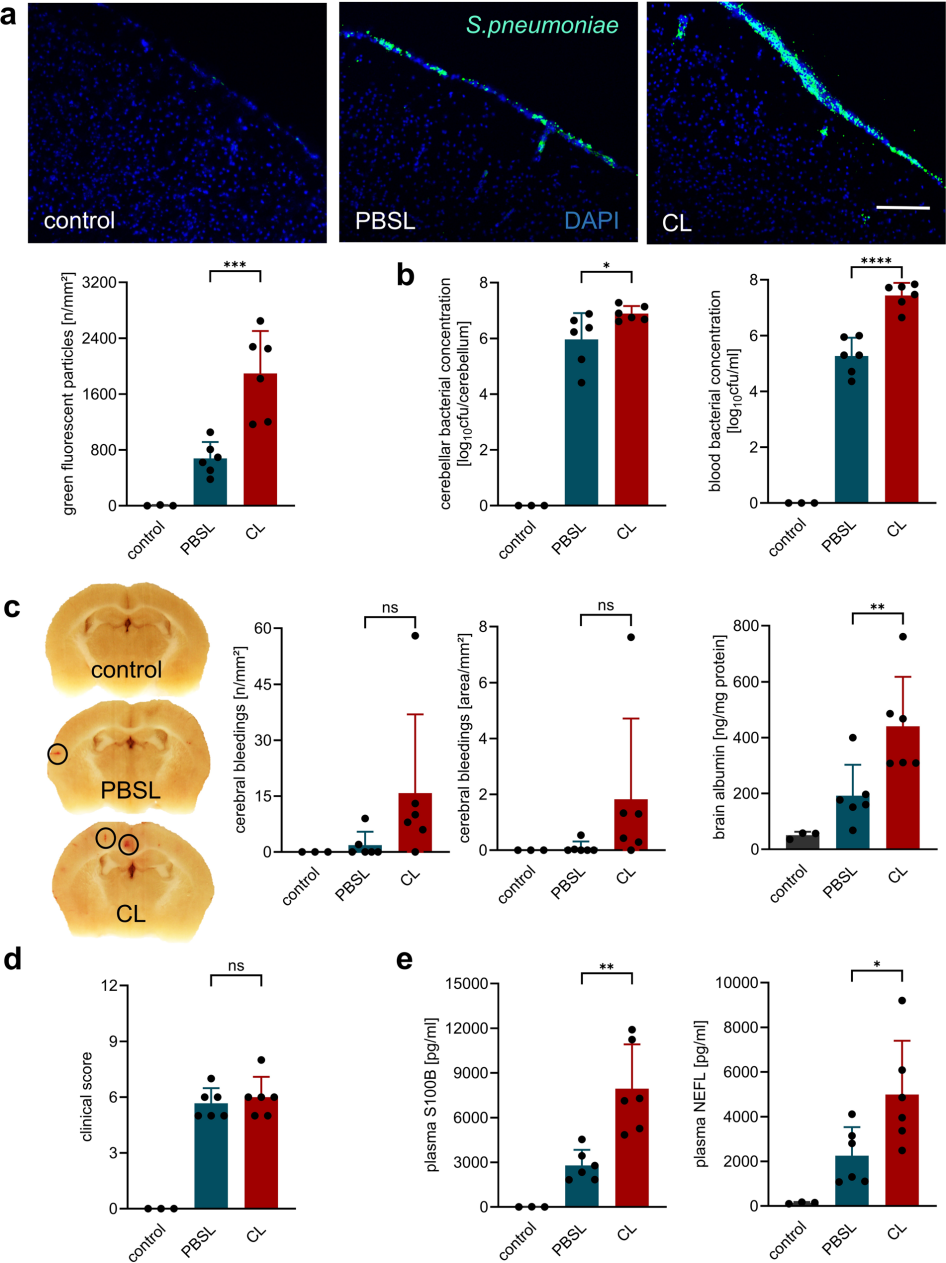
BAM depletion promotes bacterial dissemination and neuroinflammatory damage without affecting early clinical disease severity. Bacterial titers in the brain at 18 h post-infection were significantly higher in CL-treated, infected mice compared to PBSL-treated, infected controls, as evidenced by immunohistochemical staining for pneumococcal antigen (**a**, scale bar = 100 μm) as well as plating samples on Columbia blood agar plates. Titer determination by plating also revealed significantly higher bacteremia in CL-treated compared to PBSL-treated, infected mice (**b**). (**c**) Despite increased bacterial burden, no significant differences in cerebral hemorrhages were observed at this early time point (as illustrated on representative brain sections obtained from one mouse per group), while brain albumin concentrations – a marker for blood-brain barrier breakdown – were higher in CL- than in PBSL-treated, infected mice. (**d**) At this early time point, there were no significant differences in clinical scoring between both infected groups. (**e**) However, plasma levels of neurofilament light chain (NEFL) and S100B were significantly elevated in CL-treated compared to PBSL-treated, infected animals, indicating increased neuronal damage and astrocyte activation. Data represent mean ± SD; * *p* < 0.05, ** *p* < 0.01, *** *p* < 0.001, **** *p* < 0.0001 (ANOVA and Newman Keuls multiple comparison test). Since the comparison between healthy and meningitis animals served only to demonstrate disease-typical alterations, no significant differences to healthy controls are shown in the figures

As reported in previous studies [12–14], we found a significant increase in bacterial load in the blood and brain in macrophage-depleted mice, as determined by titer measurement and/or immunohistochemistry (Fig. 2). However, this did not lead to a clinically noticeable worsening of disease symptoms, although concentrations of marker molecules for neuronal damage (NEFL) and astrocyte activation (S100B) were significantly higher in the plasma of macrophage-depleted mice than in the comparison group.

Overall, our findings largely align with those of Polfliet et al. [13] in the rat model, namely that macrophage deficiency is associated with increased bacterial spread, reduced infiltration of blood leukocytes, but increased local expression of cytokines and chemokines.

### Macrophage depletion severely worsens disease progression and outcome

**Fig. 3:**
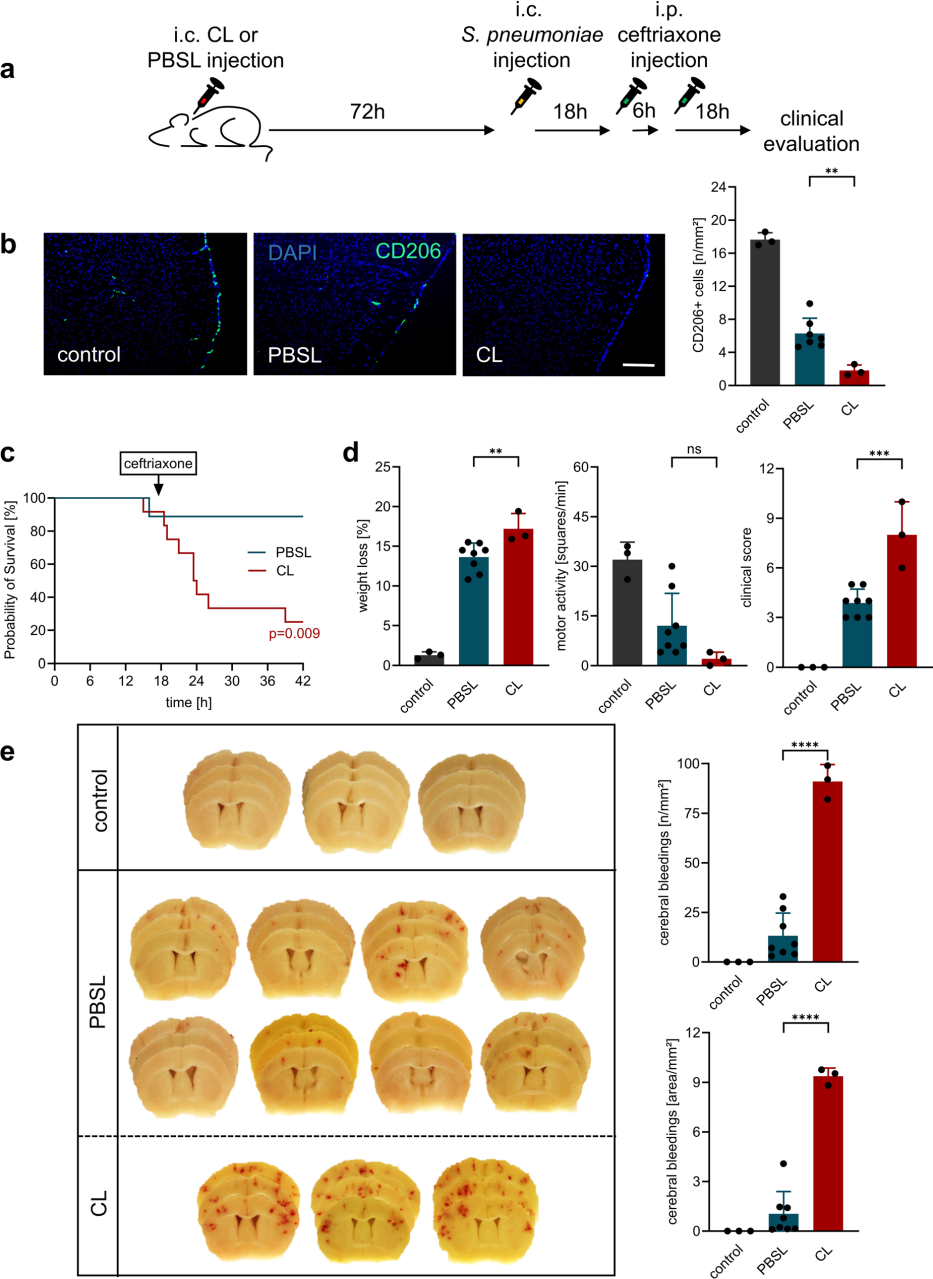
BAM depletion exacerbates clinical outcome and cerebral damage in antibiotic-treated PM. (**a**) Experimental design for extended observation period: Seventy-two hours (h) after the second intracisternal (i.c.) CL or PBSL administration, mice were infected via i.c. inoculation with live *S. pneumoniae*, treated with ceftriaxone starting 18 h post-infection, and clinically assessed 42 h post-infection (*n* = 12 and *n* = 9 per group, respectively). Control animals received only an intracisternal injection of PBS (*n* = 3) (**b**) Quantification of CD206⁺ meningeal macrophages at 42 h post-infection showed ~ 90% reduction in CL-treated infected mice, confirming still effective depletion; PM-associated reduction of this population in PBSL-treated infected mice reached ~ 65%. Scale bar = 100 μm. (**c**) Clinical outcome: Number of animals reaching humane endpoint criteria was significantly higher in CL group (9/12) vs. PBSL group (1/9). (**d**) Surviving CL-treated infected mice showed significantly greater weight loss, reduced motor activity, and higher clinical scores. (**e**) Representative images and quantification of cerebral hemorrhages revealed significantly increased number and area of hemorrhagic lesions in CL-treated infected mice compared to PBSL-treated infected mice. Data represent mean ± SD; ** *p* < 0.01, *** *p* < 0.001, **** *p* < 0.0001 (ANOVA and Newman Keuls multiple comparison test). Since the comparison between healthy and meningitis animals served only to demonstrate disease-typical alterations, no significant differences to healthy controls are shown in the figures

To further investigate the functional relevance of BAM during the later stages of PM, we extended the observation period to 42 h post-infection. To prevent overwhelming infection and associated lethality, mice were treated with the standard antimicrobial agent for PM, ceftriaxone, starting at 18 h. Even at this later time point, macrophage depletion remained effective, with a 90% and 88% reduction of CD206-positive cells in CNS border regions, namely the meninges and perivascular spaces, in CL-treated mice compared with PBSL-treated infected mice (Fig. 3). The PM-associated decline in these macrophage populations progressed further relative to the 18-hour time point, reaching approximately a 64% reduction in both populations compared with healthy controls.

This experimental approach revealed pronounced differences in disease phenotype between CL-treated and PBSL-treated meningitic mice. Only 1 of 9 PBSL-treated animals had to be euthanized due to reaching humane endpoints, whereas 9 of 12 CL-treated mice met termination criteria during the observation period. Surviving CL-treated mice exhibited more severe clinical symptoms, including greater weight loss.

**Fig. 4:**
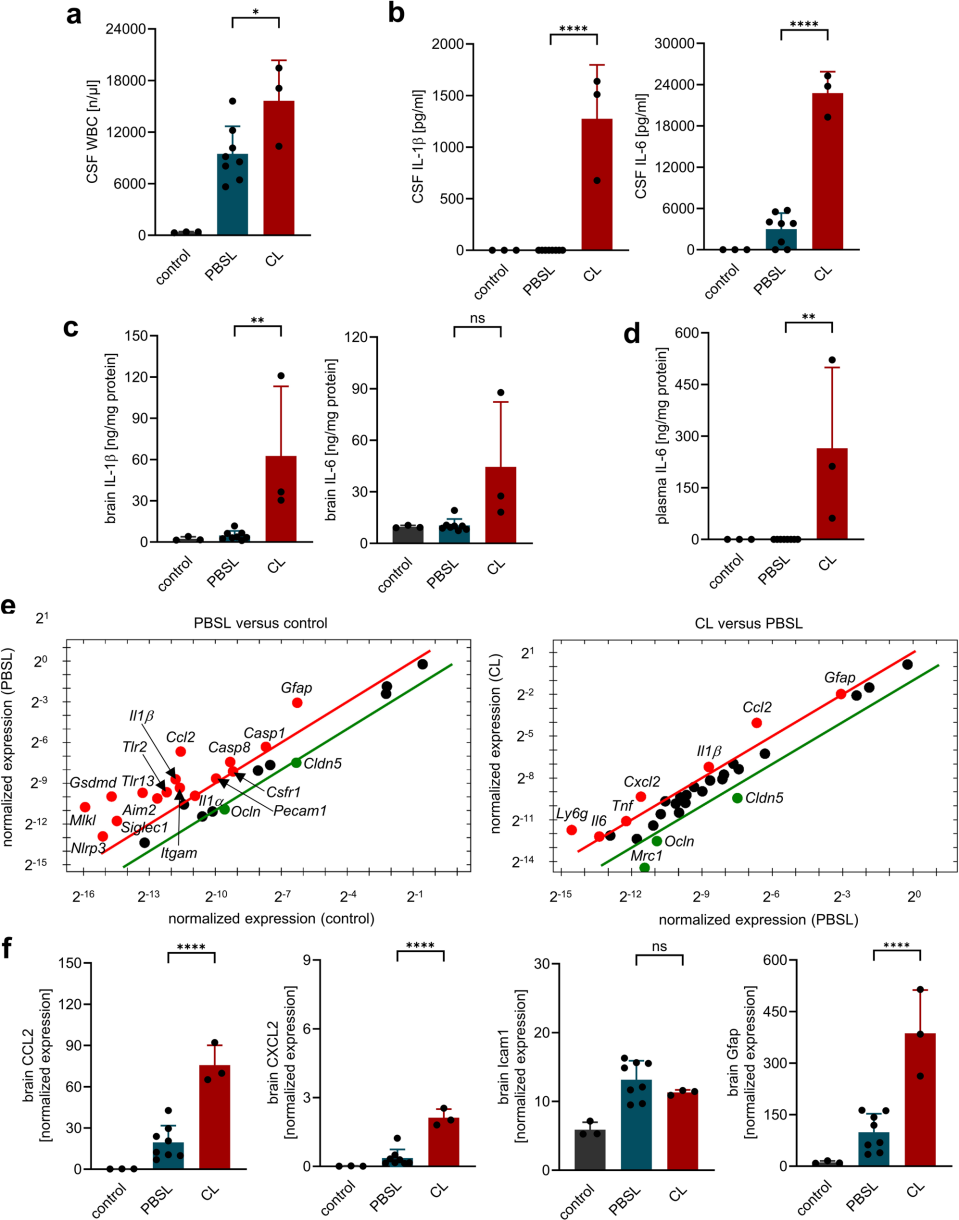
BAM depletion enhances neuroinflammation in antibiotic-treated PM. (**a**) CSF WBC counts were significantly higher in CL- than in PBSL-treated infected mice at 42 h post-infection. (**b-f**) Expression analysis (**b-d**, ELISA; **e**, PrimePCR™ array; and **f**, RT-PCR) revealed increased levels of proinflammatory cytokines and chemokines (e.g., IL-1β, IL-6, CCL2, CXCL2) and reduced expression of tight junction proteins claudin 5 (Cldn5) and occluding (Ocln) in CL-treated infected mice compared to PBSL-treated infected mice. Data represent mean ± SD; * *p* < 0.05, ** *p* < 0.01, *** *p* < 0.001, **** *p* < 0.0001 (ANOVA and Newman Keuls multiple comparison test). Since the comparison between healthy and meningitis animals served only to demonstrate disease-typical alterations, no significant differences to healthy controls are shown in the figures

Macroscopic examination of the brains revealed a highly significant increase in the number and surface area of cerebral hemorrhages in CL-treated mice compared to PBSL controls. This deterioration correlated with a persistently heightened immune response (Fig. 4): In contrast to the 18-h observation time point (see Fig. 1), CSF WBC counts were significantly higher in CL- than in PBSL-treated infected mice at 42 h post-infection.

**Fig. 5:**
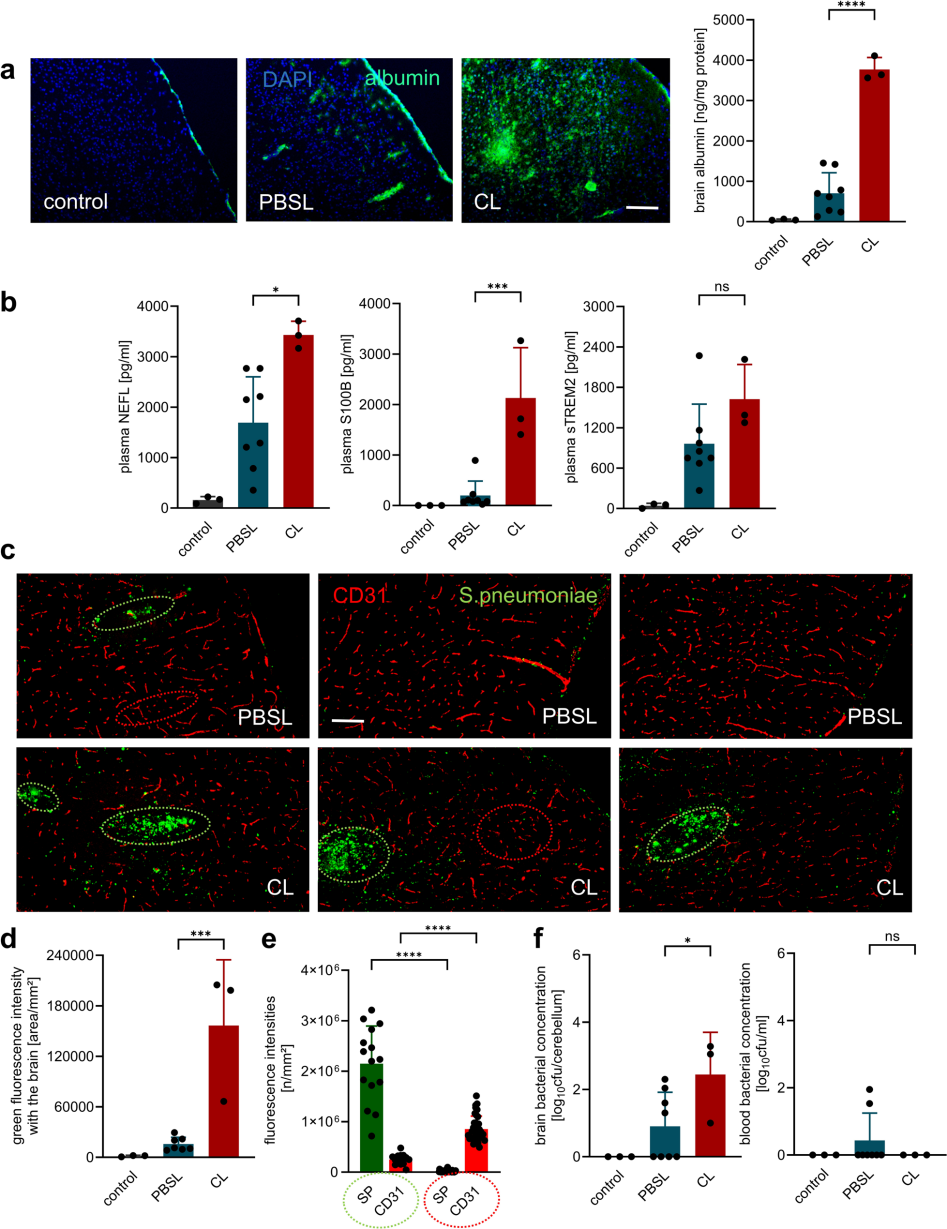
BAM depletion disrupts the blood-brain barrier and promotes bacterial dissemination in advanced PM. (**a**) Immunohistochemistry (representative images from one mouse per group) and ELISA of brain lysates (from antibiotic-treated mice sacrificed 42 h post-infection) revealed significantly increased albumin accumulation in brain tissue of CL-treated mice, indicating substantial blood-brain barrier (BBB) disruption. Scale bar = 100 μm. (**b**) Plasma concentrations of neurofilament light chain (NEFL) and S100B were significantly elevated in CL-treated animals, consistent with increased neuronal damage and astrocyte activation; levels of TREM2, a marker of microglial activation, remained unchanged. (**c, d, e**) Immunohistochemistry showed areas in the cerebral cortex with intense staining for pneumococcal antigen (marked by a green-dashed line; representative examples) in CL-treated animals. Corresponding areas exhibited markedly reduced expression of the endothelial marker CD31 compared to antigen-negative regions (marked by a red dashed line; representative examples), indicating localized vascular injury. (**f**) Bacterial titers were significantly higher in the cerebellum (but not blood) of CL- than PBSL-treated infected mice at 42 h post-infection. Data represent mean ± SD; * *p* < 0.05, ** *p* < 0.01, *** *p* < 0.001, **** *p* < 0.0001 (ANOVA and Newman Keuls multiple comparison test). Since the comparison between healthy and meningitis animals served only to demonstrate disease-typical alterations, no significant differences to healthy controls are shown in the figures

On a molecular level, CL-treated mice showed elevated brain and CSF expression of key cytokines and chemokines including IL-1β, CCL2, and CXCL2, as determined by ELISA, qPCR, and PrimePCR™ array analysis (Fig. 4**)**. This was accompanied by sustained suppression of Mrc1 (CD206), confirming effective macrophage depletion. In addition, tight junction markers claudin 5 and occludin were downregulated in CL-treated animals. Consistent with this finding, immunohistochemical analysis and ELISA of brain lysates from antibiotic-treated mice sacrificed 42 h post-infection revealed massive accumulation of albumin in the brain tissue of macrophage-depleted mice, indicating a pronounced disruption of the blood-brain barrier (BBB) in this group (Fig. 5). This was accompanied by elevated plasma concentrations of NEFL and S100B, supporting our GFAP mRNA expression data, but not of the microglial activation marker TREM2.

Investigation of bacterial burden revealed increased cerebellar titers in macrophage-depleted mice. Immunohistochemistry showed cortical areas with strong positive signals for pneumococcal antigen. In these areas, staining for the endothelial marker protein CD31 was significantly reduced compared to regions without detectable pneumococcal antigen. Together, these findings suggest that BAM play a protective role by limiting bacterial dissemination, vascular damage, and subsequent vasogenic edema during PM progression.

### Worsening of disease following macrophage depletion is driven by pneumolysin

**Fig. 6:**
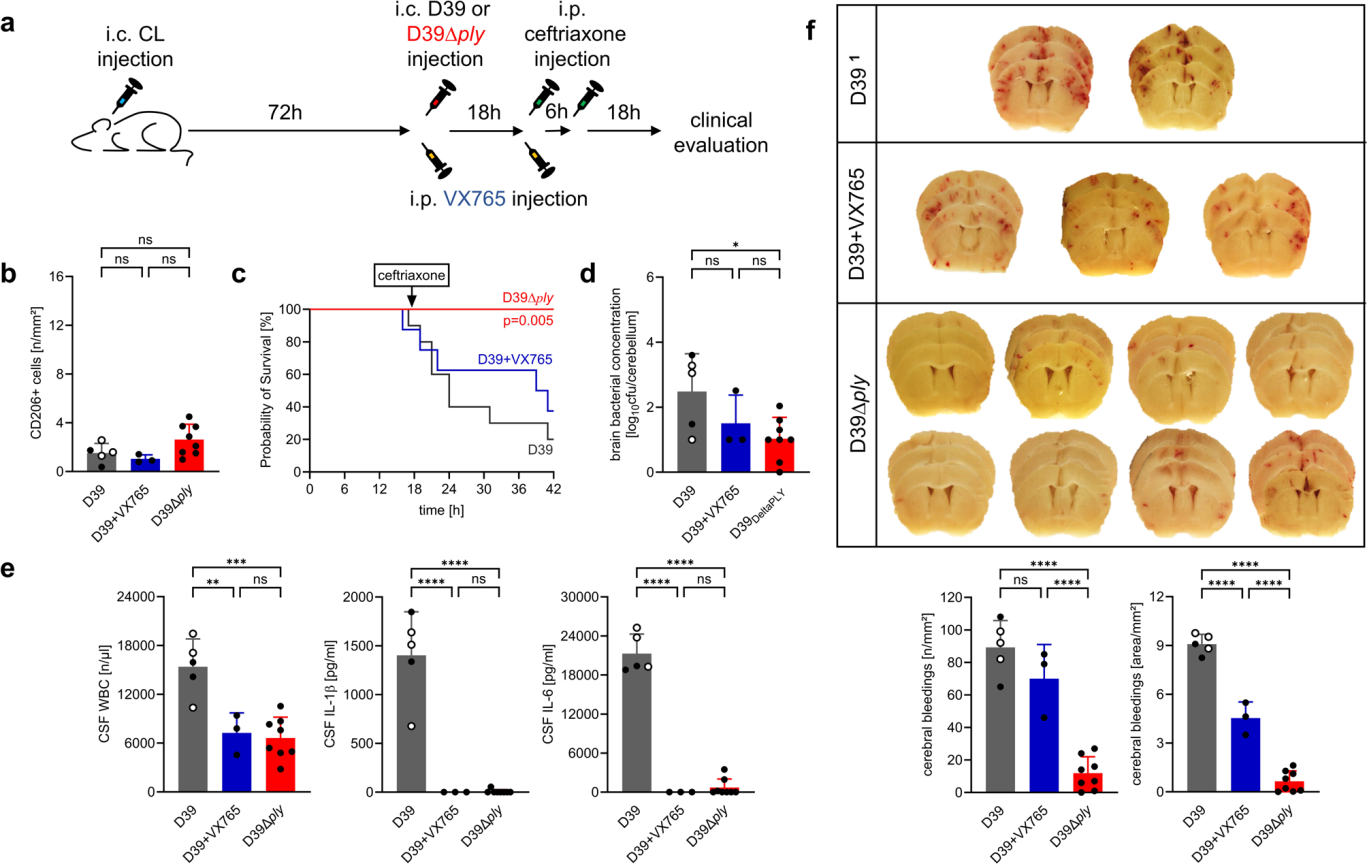
Pneumolysin, but not inflammasome signaling, mediates cerebral hemorrhages and poor clinical outcome in BAM-depleted PM. (**a**) Experimental design: Seventy-two hours (h) after CL administration, mice were either treated with the caspase-1 inhibitor VX765 and infected i.c. with wild-type *S. pneumoniae* (D39), or infected with a pneumolysin-deficient strain (D39Δply) in the absence of caspase-1 blockade (*n* = 8 per group). Eighteen h post-infection, ceftriaxone therapy was initiated, and observation continued for up to 42 h post-infection. CL-treated mice infected with D39 served as controls (*n* = 10). (**b**) Quantification of CD206⁺ meningeal macrophages at 42 h post-infection confirmed similar macrophage depletion across all groups. (**c**) All BAM-depleted mice infected with D39Δply survived, whereas 5/8 VX765-treated animals and 2/10 untreated BAM-depleted mice required euthanasia due to terminal symptoms. (**d**) Infection with D39Δply resulted in slightly lower cerebellar bacterial concentrations than with the wild type strain 42 h post-infection. (**e**) Both infection with D39Δply and treatment with VX765 were associated with attenuated brain inflammation, as indicated by lower CSF WBC counts and reduced CSF IL-1β and IL-6 levels. (**f**) Representative brain sections and quantification of hemorrhagic lesions revealed a slight reduction in hemorrhages in VX765-treated mice, but a significantly decreased number and area of hemorrhages in mice infected with D39Δply, reaching levels comparable to PBSL-treated wild-type controls (see Fig. 3). ^1^Representative brain sections from the other three mice with the same treatment regimen are shown in Fig. 3 (designated as the ‘CL’ group). In graphs **b**, **d**, **e**, and **f**, pooled data from both experimental series with CL-treated infected mice are shown (first series: open circles; second series: filled black circles) and were used for statistical analysis. Data represent mean ± SD; * *p* < 0.05, ** *p* < 0.01,*** *p* < 0.001, **** *p* < 0.0001 (ANOVA and Newman Keuls multiple comparison test)

To determine whether the worsened clinical outcomes observed in macrophage-depleted mice were primarily due to a sustained hyperinflammatory response or to increased bacterial burden and associated cytotoxicity, we conducted further experiments in macrophage-depleted animals. One experimental group received the caspase-1 inhibitor VX765, as the inflammasome-dependent signaling pathway is a key driver of the exaggerated immune response in PM [18–21]. A second group was infected not with the wild-type strain (D39), but with an isogenic pneumolysin-deficient mutant of D39 (D39Δ*ply*) [22–24]. There were no significant differences between groups in terms of BAM macrophage depletion. (Fig. 6). However, the clinical outcomes differed markedly: all BAM-depleted mice infected with the pneumolysin-deficient strain survived, whereas 5 of 8 VX765-treated and 8 of 10 untreated, infected mice had to be euthanized due to the development of terminal symptoms. In line with these clinical findings, anti-inflammatory treatment with VX765 resulted in only a minor reduction in the number and area of cerebral hemorrhages. Hemorrhagic lesions in BAM-depleted mice infected with the pneumolysin-deficient mutant were comparable to those in PBSL-treated controls infected with the wild-type strain (see Fig. 3), indicating a significant reduction relative to wild type-strain-infected, BAM-depleted mice. Notably, both infection with the pneumolysin-deficient strain and treatment with VX765 were associated with attenuated brain inflammation, as reflected by lower CSF WBC counts and reduced CSF IL-1β and IL-6 levels, whereas cerebellar bacterial concentrations were slightly lower in mice infected with the pneumolysin-deficient strain but not in those treated with VX765 (Fig. 6). These findings suggest that the increased neuropathology and worsened outcome in macrophage-depleted mice are primarily attributable to enhanced bacterial cytotoxicity mediated by pneumolysin, rather than to excessive host inflammation alone.

## Discussion

Our findings refine the understanding of the role of resident BAM in PM, particularly with respect to immune activation and containment of tissue injury. Contrary to the prevailing concept [10, 11] that these cells are indispensable initiators of the host immune response, our data - together with a careful reevaluation of previous studies [13, 14] - indicate that they do not represent major sources of cytokines and chemokines, nor do they play a predominant role in driving leukocyte infiltration during the early phase of PM. Rather, their primary function may lie in limiting pneumococcal outgrowth as well as the magnitude and duration of the inflammatory response, thereby protecting the structural integrity of the brain and its border zones.

Importantly, we observed a significant, natural reduction in the number of CD206-positive BAM in meningitic mice as the infection progressed. This depletion occurred independently of CL treatment and likely reflects in situ cell death, or less likely, egress under inflammatory conditions, given that BAM are generally considered to be sessile [25–28]. A similar loss of resident macrophages during CNS infections has been reported in mouse models of intracranial lymphocytic choriomeningitis virus [29], intravenous group B streptococcal infection [30], and a zebrafish embryo model of *S. pneumoniae* hindbrain ventricle infection [31], suggesting that macrophage attrition may be an intrinsic feature of meningitis progression - potentially impairing local regulation of inflammation. However, the functional consequences of this endogenous loss remain largely unexplored. Our data suggest that when the macrophage compartment falls below a critical threshold - whether due to experimental depletion or disease-associated loss - this results in an amplified and sustained inflammatory response, associated with poorer clinical outcomes.

In light of our current data, and building on previous reports, we propose that resident BAM play a modulatory rather than an initiatory role in PM. They do not appear to be the primary drivers of cytokine and chemokine production or leukocyte recruitment, as these responses persist - and in some aspects even intensify - in their absence. Instead, the primary function of BAM in PM seems to be the guidance and regulation of the host immune response to the pathogen, thereby preventing excessive activation of inflammatory cascades and limiting secondary tissue damage, particularly beyond the acute phase of infection once effective antimicrobial therapy has started. This interpretation is supported by our observation of persistently elevated levels of IL-1β, IL-6, CCL2, and CXCL2, as well as sustained CSF pleocytosis in CL-treated mice despite ongoing antimicrobial treatment. These findings align well with a recently proposed concept suggesting that BAM primarily fulfill trophic functions that support tissue homeostasis, rather than acting as immune sentinels essential for border defense. In contrast, blood-borne phagocytes - predominantly neutrophilic granulocytes in PM [32, 33] - are the main drivers of immunopathology [11, 28, 34].

The consequences of macrophage depletion were particularly striking at the vascular interfaces, where key immune interactions occur during PM. We observed significant vascular dysfunction, including widespread cerebral hemorrhages and increased BBB disruption in CL-treated meningitic mice. The latter was reflected by the downregulation of tight junction proteins (claudin 5, occludin) and the accumulation of blood-derived albumin in brain tissue, likely resulting from both inflammatory signaling and direct bacterial interactions with the vasculature. These findings underscore the critical role of meningeal and perivascular macrophages in maintaining barrier function and vascular integrity in PM. Consistently, the involvement of these macrophage subsets in vascular homeostasis has been proposed in recent review articles [28, 34], based on the following observations: (i) genes universally upregulated in these macrophage populations are associated with blood vessel development [35]; (ii) perivascular macrophages, together with laminin-containing basement membranes, form a vascular barrier in the rat area postrema [36]; (iii) perivascular macrophages - distinct from microglia - contribute to the maintenance of the blood-retinal barrier under both physiological and pathological (retinopathy) conditions [37]; and (iv) an intact monocyte/macrophage population is required to restore BBB integrity following traumatic brain injury [38]. Notably, other experimental studies have implicated perivascular macrophages in BBB or blood-spinal barrier breakdown under pathological conditions, including amyotrophic lateral sclerosis [39], hypertension [40], and acute cerebral ischemia [41]. Collectively, these findings suggest that BAM play a context- and tissue-specific role in regulating vascular integrity, supporting the need for further targeted experimental investigations.

Insights into the mechanisms underlying aggravated vascular injury in the absence of macrophages were gained from our complementary experiments using a pneumolysin-deficient pneumococcal strain and the inflammasome inhibitor VX765. Whereas VX765 was ineffective, animals infected with the pneumolysin-deficient strain were protected from severe clinical deterioration and hemorrhagic complications. This suggests that macrophage depletion creates a permissive environment for bacterial outgrowth and dissemination, as demonstrated by a stronger immunofluorescence signal for pneumococcal antigen in the leptomeningeal space and, even more strikingly, within the superficial cerebral cortex (Figs. 2 and 5). As a consequence of macrophage depletion, bacterial toxins - particularly pneumolysin - are released at higher concentrations and at ectopic sites, especially around intraparenchymal cerebral vessels (distant from the leptomeningeal space), leading to increased vascular injury and disease exacerbation. The cytotoxic activity of pneumolysin on endothelial cells is well established, both in cell culture experiments [42–45] and in animal models, particularly of pneumococcal pneumonia [46, 47]. Notably, the impact of pneumolysin deficiency on disease phenotype in our study is far greater than previously reported, where only modestly milder symptoms and moderate effects on inflammation were observed - however, these studies were conducted in the absence of antimicrobial therapy [19, 48–52]. In contrast, caspase-1 inhibition - previously shown to be protective in PM models with intact macrophages [18, 19, 53, 54] - provided little or only partial protection in our setting. These results indicate that under conditions of high bacterial load with concurrent antibiotic treatment, bacterial toxins - particularly pneumolysin - are the main drivers of brain injury, whereas hyperinflammation plays a relatively minor role. This interpretation is further supported by clinical studies linking high bacterial burden and elevated pneumolysin levels to poor outcomes [55, 56].

Our findings also help reconcile discrepancies in earlier studies [12–14]. Differences in timing, species, and experimental design likely contributed to varying conclusions about the role of BAM. By extending our observation period beyond the early phase and analyzing both molecular and anatomical outcomes, we revealed a stage-dependent function of these cells that is missed in short-term analyses.

A limitation of our study is the potential for off-target effects of CL. Although local administration (e.g., intracisternal, i.c.) of CL is widely considered an effective method for niche-specific macrophage depletion with minimal systemic side effects [17], off-target effects cannot be entirely ruled out. Other phagocytic cell populations, such as neutrophils and mast cells, may also be affected [57]. Supporting this, systemic (i.v.) administration of CL has been shown to deplete not only mononuclear phagocytes but also to impair the function of polymorphonuclear neutrophils [58]. However, as neutrophils are key effector cells mediating brain injury in PM, their inactivation is unlikely to explain the observed effects in our model, particularly since CL treatment led to disease exacerbation rather than improvement. Moreover, we evaluated the efficacy of CL administration using a single approach, namely the reduction of CD206-positive cells. This approach is supported by numerous studies identifying CD206 as a reliable marker for BAM [35, 59–61]. Furthermore, CD206 detection was used in a key previous study investigating the effects of macrophage depletion in a mouse model of hematogenous PM [12]. However, this method alone does not provide a comprehensive assessment of CL treatment effects on the diverse BAM subpopulations, which can differ considerably in their CD206 expression levels.

Another limitation is that we used the model most commonly employed in meningitis research, in which the pathogens are directly injected into the cisterna magna. This model reflects direct infection from a neighboring infectious focus - accounting for 40–50% of PM cases in adult patients [7, 62] - but does not mimic the hematogenous origin of meningitis. In a hematogenous PM model, *S. pneumoniae*-activated dural nociceptors have been shown to suppress macrophage chemokine expression, neutrophil recruitment and dural antimicrobial defences through CGRP-RAMP1 signaling, thus facilitating bacterial brain invasion; however, this effect was not observed following i.c. inoculation [12]. Notably, i.c. administration of CL increased bacterial loads but reduced leukocyte infiltration in rodent PM, irrespective of the bacterial inoculation route [12, 13] (this study). Further studies in the intravenous model are warranted to assess whether our observations in the i.c. model - particularly concerning the immunosuppressive and tissue-protective functions of BAM - can be recapitulated in the intravenous model or if they exhibit model-specific differences.

A further limitation is that the exact mechanisms by which macrophages contribute to vascular “stabilization” remain unclear. These effects may primarily involve macrophage-mediated control of bacterial replication and outgrowth, ultimately leading to reduced pneumolysin release. Given that pneumolysin is a key virulence factor associated with cerebral hemorrhages and adverse outcomes, its increased release in the absence of macrophages could explain the observed vascular damage and worsened disease progression in macrophage-depleted mice.

In conclusion, our results challenge the traditional view of BAM as primarily pro-inflammatory effectors. Instead, they play a critical immunoregulatory role during the progression of PM, limiting inflammation and/or promoting its resolution, preserving vascular integrity, and protecting against tissue injury. Loss of these cells - whether due to disease progression or experimental depletion - results in prolonged inflammation, enhanced pneumococcal dissemination with subsequent toxin release, and vascular damage, underscoring their importance as guardians of CNS homeostasis in bacterial meningitis. These findings suggest that optimal therapeutic strategies may require not only early and effective antibiotic treatment to restrict bacterial proliferation, but also preservation of the BAM compartment.

## Supplementary Information

Below is the link to the electronic supplementary material.

Supplementary Material 1

Supplementary Material 2

## Data Availability

The datasets used and/or analysed during the current study are available from the corresponding author on reasonable request.

## Abbreviations

ANOVA: Analysis of variance
BAM: Border-associated macrophages
BSA: Bovine serum albumin
CCL2: CC-chemokine ligand 2
CFU: Colony-forming units
CL: Clodronate liposomes
CNS: Central nervous system
CSF: Cerebrospinal fluid
CXCL2: CXC motif chemokine ligand 2
DALYs: Disability-adjusted life years
GFAP: Glial fibrillary acidic protein
GAPDH: Glyceraldehyde-3-phosphate dehydrogenase
h: Hour(s)
i.c.: Intracisternal
ICAM1: Intercellular adhesion molecule 1
IL: Interleukin
i.p.: Intraperitoneal
Mrc1 (CD206): Mannose receptor c-type 1
NEFL: Neurofilament light chain
OFT: Open field test
PBS: Phosphate-buffered saline
PBSL: Phosphate-buffered saline liposomes
PECAM1 (CD31): Platelet and endothelial cell adhesion molecule 1
PLY: Pneumolysin
PM: Pneumococcal meningitis
RT: Room temperature
S. pneumoniae: Streptococcus pneumoniae
S100B: S100 calcium binding protein B
SD: Standard deviation
TREM2: Triggering receptor expressed on myeloid cells 2
WBC: White blood cells
