# Supplementary material for: Pharmacologic depletion of border-associated macrophages worsens disease in a mouse model of meningitis
Source: Acta Neuropathol Commun. 2025 Sep 23;13:191. doi: 10.1186/s40478-025-02126-5 (PMC12455799; doi:10.1186/s40478-025-02126-5)
Supplement: Supplementary file 1 — Supplementary Material 1 [file 40478_2025_2126_MOESM1_ESM.pdf]

Supplemental table: list of elements on the customized PrimePCR Array

Gene code	Protein
Aim2	AIM2, absent in melanom 2
Casp1	CASP1, caspase 1
Casp3	CASP3, caspase 3
Casp8	Casp8, caspase 8
Ccl2	CCL2 , C-C motif chemokine 2
Ccr2	CCR2, C-C Motif Chemokine Receptor 2
Cldn5	CLDN5, claudin 5
Csfr1	CSFR1, colony stimulating factor 1 receptor
Cxcl2	CXCL2, C-X-C motif chemokine 2, growth-regulated protein beta
Gfap	GFAP, glial fibrillary acidic protein
Gsdmd	GSDMD, gasdermin D
Il1a	IL-1α, interleukin-1beta
Il1b	IL-1β, interleukin-1beta
Il6	IL-6, interleukin-6
Itgam	ITGAM, integrin subunit alpha M, CD11B
Ly6g	LY6G, lymphocyte antigen 6 complex locus G6D
Mif	MIF, macrophage migration inhibitory factor
Mlkl	MLKL, mixed lineage kinase domain like pseudokinase
Mrc1	MRC1, macrophage mannose receptor 1, CD206
Nefl	NEFL, neurofilament light polypeptide
Nlrp3	NLRP3, NACHT, LRR and PYD domains-containing protein 3
Ocln	OCLN, occludin
Pdgfrb	PDGFRβ, platelet-derived growth factor receptor alpha
Pecam1	PECAM1, platelet and endothelial cell adhesion molecule 1, CD31
Siglec1	SIGLEC1, sialic acid binding Ig like lectin 1, CD169
Tgfb2	TGFβ_2, transforming growth factor beta 2

Tlr13	TLR2, Toll-like receptor 13
Tlr2	TLR2, Toll-like receptor 2
Tmem19	TMEM19, transmembrane protein 19
Tnf	TNF, tumor necrosis factor
*Actb**	ActB, actin, cytoplasmic 1, beta-actin
*Gapdh**	GAPDH, glyceraldehyde-3-phosphate dehydrogenase

* housekeeping genes
