# Supplementary figures and images for: Pharmacologic depletion of border-associated macrophages worsens disease in a mouse model of meningitis
Source: Acta Neuropathol Commun. 2025 Sep 23;13:191. doi: 10.1186/s40478-025-02126-5 (PMC12455799; doi:10.1186/s40478-025-02126-5)

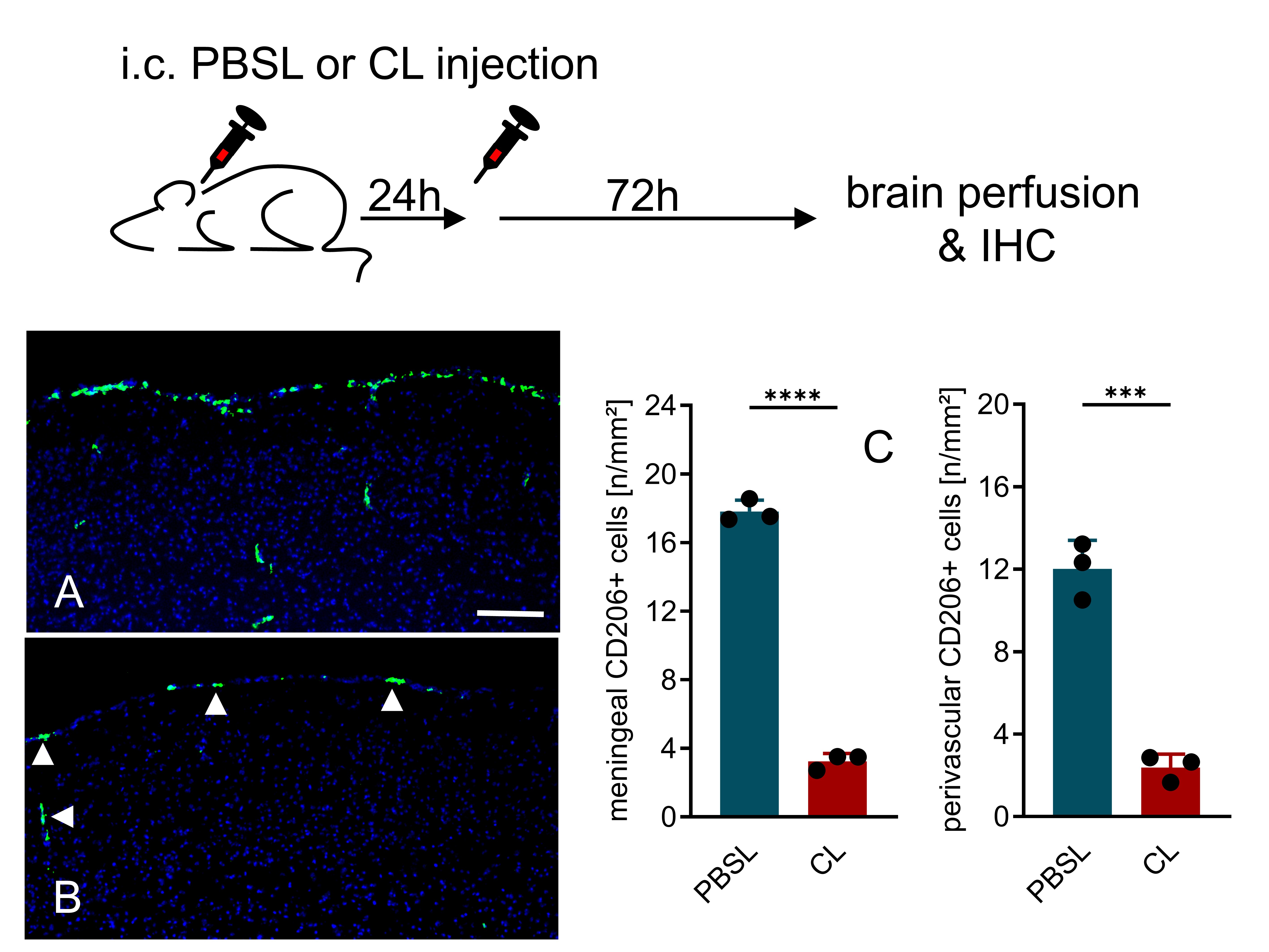

Supplement: Supplementary file 2 — Supplementary Material 2 [file 40478_2025_2126_MOESM2_ESM.jpg]
